# Phytochemical Profile and Functional Properties of the Husk of *Argania spinosa* (L.) Skeel

**DOI:** 10.3390/plants14152288

**Published:** 2025-07-24

**Authors:** Antonietta Cerulli, Natale Badalamenti, Francesco Sottile, Maurizio Bruno, Sonia Piacente, Vincenzo Ilardi, Rosa Tundis, Roberta Pino, Monica Rosa Loizzo

**Affiliations:** 1 Department of Pharmacy, University of Salerno, 84084 Fisciano, Italy; acerulli@unisa.it (A.C.); piacente@unisa.it (S.P.); 2 Department of Biological, Chemical and Pharmaceutical Sciences and Technologies (STEBICEF), Università degli Studi di Palermo, Viale delle Scienze, Ed. 17, 90128 Palermo, Italyvincenzo.ilardi@unipa.it (V.I.); 3 National Biodiversity Future Center (NBFC), 90133 Palermo, Italy; 4 Department of Architecture, University of Palermo, Viale delle Scienze, 90128 Palermo, Italy; francesco.sottile@unipa.it; 5 Centro Interdipartimentale di Ricerca “Riutilizzo Bio-Based Degli Scarti da Matrici Agroalimentari” (RIVIVE), University of Palermo, Viale delle Scienze, 90128 Palermo, Italy; 6 Department of Pharmacy, Health and Nutritional Sciences, University of Calabria, 87036 Rende, Italy; rosa.tundis@unical.it (R.T.); roberta.pino@unical.it (R.P.); monica_rosa.loizzo@unical.it (M.R.L.)

**Keywords:** *Argania spinosa*, Sapotaceae, LC-ESI/HRMSMS analysis, by-products, antioxidant activity, hypoglycaemic and hypolipidemic effect

## Abstract

Due to the limited scientific exploration of *Argania spinosa* (L.) skeel husk, this study presents the first investigation of the metabolite profile of methanol and acetone extracts analyzed by liquid chromatography coupled with electrospray ionization and high-resolution multistage mass spectrometry (LC-ESI/HRMSMS). A total of 43 compounds, including hydroxycinnamic acid and flavonoid derivatives, saponins, and triterpenic acids, were identified, some of which have not been previously reported in this species. The total phenols (TPC) and flavonoids (TFC) content were spectrophotometrically determined. A multi-target approach was applied to investigate the antioxidant potential using 1,1-Diphenyl-2-picrylhydrazyl (DPPH), 2,2-azino-*bis*-3-ethylbenzothiazoline-6-sulphonic acid (ABTS), *β*-carotene bleaching, and Ferric Reducing Ability Power (FRAP) tests. Carbohydrate hydrolyzing enzymes and lipase inhibitory activities were also assessed. The acetone extract exhibited the highest TPC and TFC values, resulting in being the most active in *β*-carotene bleaching test with IC_50_ values of 26.68 and 13.82 µg/mL, after 30 and 60 min of incubation, respectively. Moreover, it was the most active against both α-glucosidase and α-amylase enzymes with IC_50_ values of 12.37 and 18.93 µg/mL, respectively. These results pointed out that this by-product is a rich source of bioactive phytochemicals potentially useful for prevention of type 2 diabetes and obesity.

## 1. Introduction

Argan tree is a woody species belonging to the Sapotaceae family and represents a tree endemic exclusively to southwestern Morocco, where it forms a unique ecosystem. In this environment, it plays a crucial role in maintaining biodiversity balance as well as supporting the socioeconomic livelihoods of local communities [1].

Several ethnobotanical and ethnopharmacological studies have documented the traditional use of various parts of the plant, highlighting its wide range of uses and applications [2,3]. Local populations use the fruits, seeds, pulp, leaves, bark, wood, and roots of *A. spinosa* to treat pathological conditions such as diabetes, rheumatism, eczema, burns, hypercholesterolemia, gastritis, ulcers, dysentery, headache, fever, and various dermatological and hair-related disorders [4,5].

The dried seeds are applied externally in powder form to treat eczema, sprains, skin lesions, and burns [3,6], as well as for hair care [7].

This species has increasingly attracted international commercial interest due to the oil extracted from its seeds—known as argan oil—the valorization of which has helped reactivate productive and employment dynamics in rural areas of Morocco. Argan oil is characterized by a lipid profile rich in unsaturated fatty acids, tocopherols, and phytosterols, which make it suitable for diverse applications. Traditionally, it forms part of the Moroccan diet and is appreciated for its nutraceutical properties and distinctive nutty flavor [8]. Its cosmetic applications include skin hydration and the treatment of scars and acne [9].

The nutritional, antioxidant, and dermo cosmetic properties of argan oil, together with its growing presence in international markets, have determined the need for in-depth studies concerning the ripening stage of the fruit, harvesting methods, storage conditions, and extraction processes, all of which significantly affect its quality [10]. In recent years, mechanized extraction processes have been developed, enabling the production of microbiologically safe, stable, and organoleptically acceptable oil [1].

Regarding the by-products of argan processing, argan press cake—the solid residue obtained after seed pressing—is sometimes used as a livestock feed supplement, often combined with the plant’s leaves. The fruit pulp is used in leather tanning and, in some local traditions, also in topical applications [1,5].

Research focused on the use of the *Argania spinosa* husk—the part commonly referred to as the shell or outer covering of the seed—remains limited or superficial. This component is often overlooked in the argan oil production chain. However, recent studies on other nut species suggest potential applications of this by-product in various fields [11], starting with agronomic uses as a soil amendment. It can significantly contribute to improved water retention capacity and soil fertility. Furthermore, it supports the incorporation of organic matter, enhancing beneficial microbial activity essential for soil and crop health.

The presence of phenolic and antioxidant compounds in the husk is still not well documented in argan, unlike in other nut skins [11]. Isolating and characterizing such compounds could pave the way for new applications in the nutraceutical sector as functional ingredients in dietary supplements or fortified foods [11]. The husk may also serve as an ingredient in dermocosmetic products and promote cellular regeneration.

These applications provide value to an otherwise discarded by-product, contributing to the sustainability of the argan value chain and fostering the development of necessary circular economy policies. In this way, the valorization of the argan husk not only reduces waste in the production chain but may also support the development of innovative and sustainable products, and, in particular, antioxidant products. Indeed, several phenolic compounds, extracted from waste, have been demonstrated to possess significant antioxidant potential [12,13]. Some of these compounds have been identified in *Argania spinosa* leaves, highlighting their capacity as antidiabetic and antioxidant agents [14,15].

Based on the limitation of scientific data on *Argania spinosa* husk, the aim of this study was to investigate the metabolite profile of methanol (CH_3_OH) and acetone (CH_3_)_2_CO extracts using ultra-high-performance liquid chromatography coupled to hybrid quadrupole-Orbitrap mass spectrometer, using negative electrospray ionization mode, in tandem mass spectrometry mode [UHPLC-(−)ESI/Q Exactive MS/MS] as well as the *in vitro* biological potential of this by-product as antioxidant and antidiabetic agent.

## 2. Results and Discussion

### 2.1. Chemical Composition of Argania spinosa Husk Extracts

**Figure 1:**
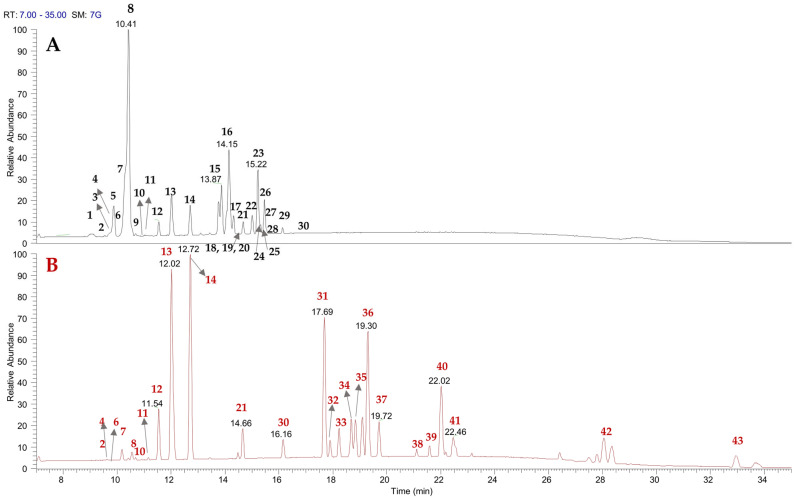
LC-ESI/HRMSMS profiles of *A. spinosa* husk methanol (**A**) and acetone extracts (**B**).

Previous studies have highlighted that the *A. spinosa* kernel is rich in phenolics, flavonoids, and saponins [16,17,18,19,20]. The CH_3_OH and (CH_3_)_2_CO extracts of *A. spinosa* husk have been analyzed using UHPLC-(−)ESI/Q Exactive MS/MS. By carefully examining accurate mass measurements, fragmentation patterns, and cross-referencing with literature data, 43 metabolites have been identified. These were categorized into different chemical classes, including hydroxycinnamic acid derivatives (**2**, **3**, **6**, **7**), flavanol (**1**, **4**, **5**, **8**, **9**) and flavonol (**10**–**14**, **19**, **21**, **30**) derivatives, saponins (**15**–**18**, **20**, **22**–**29**), and triterpenic acids (**31**–**43**) (Figure 1 and Table 1).

Careful analysis of the LC-HRMS spectra suggested the presence of the flavanols catechin (**4**) and epicatechin (**8**), as confirmed by comparison with standards, along with their procyanidin derivatives—procyanidin B1 (**1**), procyanidin B2 (**5**), and procyanidin C1 (**9**)—as supported by key product ions in the LC-HRMS/MS spectra and by literature data. Indeed, these compounds have been previously reported in *A. spinosa* fruits [16]. Furthermore, the analysis of flavonoid derivatives enabled the identification of flavonol derivatives and revealed the presence of one or more sugar moieties linked to the aglycone. In detail, compounds **10**–**14**, and **19** displayed characteristic fragmentation patterns, with base peaks indicating the neutral loss of sugar units such as hexose (162 Da), deoxyhexose (146 Da), or pentose (132 Da). In addition, the above-mentioned compounds exhibited a base peak at *m*/*z* 301, corresponding to the molecular formula C_15_H_9_O_7_, consistent with [quercetin-H]^−^. Compound **30** was unequivocally identified as the aglycone quercetin using the standard. Even if the occurrence of compounds **1**, **4**, **5**, **8**–**14**, **19**, **21**, and **30** has been documented in *A. spinosa* fruit, this is the first report of their presence in the husk [16,17].

Peaks **2**, **3**, **6**, and **7** displayed MS/MS fragmentation patterns typical of hydroxycinnamic acid derivatives. They exhibited a neutral loss of 180 Da, corresponding to a hexose moiety, indicating the presence of glycosylated derivatives, along with product ions characteristic of hydroxycinnamic acid linked to hexose. Specifically, the product ions at *m*/*z* 145.0283, 161.0340, 175.0390, and 205.0497 corresponded to the mono-dehydrated forms of coumaroyl, caffeoyl, feruloyl, and sinapoyl units in compounds **2**, **3**, **6**, and **7**, respectively [21].

LC-HRMS/MS analysis of CH_3_OH extract enabled the identification of compounds **15**–**18**, **20**, and **22**–**29** as saponins. Fragmentation data from these saponins provided insights into the type of aglycone and the number of sugar units. The majority of saponins revealed in the methanol extract of *A. spinosa* were derived from protobassic acid (compounds **22**, **23**, **24**, **26**, **28**, and **29**) or 16α-hydroxyprotobassic acid (compounds **15**–**18**, and **20**). Minor saponins derived from other sapogenins, such as bayogenin (compounds **25** and **27**), were also detected. All mentioned saponins—except compound **20**—have been previously identified in *A. spinosa* kernel [22,23]. However, only compounds **15**, **22**, and **26** have been previously reported in the husk of argan fruit [24]. Consequently, compounds **16**–**18**, **20**, **23**–**25**, and **27**–**29** are reported here for the first time in *A. spinosa* husk.

LC-HRMS/MS allowed the assignment of compounds **31**–**43**, detected only in (CH_3_)_2_CO extract, as triterpenic acids. In particular, compounds **32**, **34**, and **40** were identified as the aglycones of arganines, 16-α-hydroxyprotobassic acid, protobassic acid, and bayogenin, respectively; while compounds **37** and **41** were assigned as protobassic acid and bayogenin isomers, respectively. The base peaks and LC-HRMS/MS spectra of compounds **31**, **33**, **35**, **36**, **38**, and **39** allowed their tentative assignment as triterpenic acids chemically related to the aforementioned aglycones, with slight modifications. Compounds **31**–**41** have never been reported in *A. spinosa*. Notably, the presence of zanhic acid (**31**) and medicagenic acid (**33**), typical aglycones of *Medicago sativa* saponins [22], is here reported for the first time in *A. spinosa*. Finally, compounds **42** and **43** were identified as maslinic and oleanolic acid, respectively, which have been previously reported in *A. spinosa* fruits [18], but never in argan husk.

Among the analyzed extracts, flavonoids and most hydroxycinnamic acid derivatives were present in both extracts; saponins were mainly found in the CH_3_OH extract, and triterpenic acids were detected exclusively in the (CH_3_)_2_CO extract, reflecting the polarity of the solvents used for extraction.

### 2.2. Antioxidant Activity of Argania spinosa Husk Extracts

Determination of Total Phenols Content (TPC) and Total Flavonoids Content (TFC) revealed that (CH_3_)_2_CO extract exhibited the highest bioactive content with values of 20.42 mg CAE/g dry extract and 12.7 mg QE/g dry extract, respectively, whereas values of 7.62 mg CAE/g dry extract and 1.78 mg QE/g dry extract were detected for CH_3_OH extract.

The analysis of antioxidant potential was carried out using a multi-target approach. Samples exhibited antioxidant activity in a concentration-dependent manner. Methanol extract resulted in the most active in both radical scavenging activity test with IC_50_ values of 198.34 and 302.23 µg/mL for DPPH and ABTS test, respectively, (Table 2, Figures S1a and S2a).

The same sample exhibited a promising FRAP activity with a value comparable to that of the positive control BHT (54.88 vs. 63.44 μM Fe (II)/g). The β-carotene bleaching assay investigates the ability of the antioxidants to inhibit lipid peroxidation in the phase of initiation as well as in the phase of propagation [25]. As shown in Figure S1a and b, both extracts exhibited a promising protective effect in a concentration-dependent manner.

Previously, El Monfalouti et al. [24] investigated the content of bioactive compounds and antioxidant potential of shell and kernels from argan. Authors found a TPC of 8.2 mg GAE/g in kernels. These samples exhibited ABTS radical scavenging potential. More recently, Mirpoor et al. [25] confirmed the antioxidant effect of argan seed oil cakes. Methanolic extracts from seed and kernel obtained from *A. spinosa* from Morocco were investigated for their total phytochemical content and bioactivity. TPC values of 49.36 and 207.52 mg GAE/g extract for seeds and kernels, respectively, were found, whereas values of 18.41 and 103.43 mg QE/g extract were detected in the same samples for TFC. Notably, no DPPH or ABTS scavenging effect was found in argan seed, whereas kernels exhibited a promising radical scavenging potential with IC_50_ values of 11.69 and 62.51 µg/mL for DPPH and ABTS, respectively, [26]. On the contrary, pulp methanol extract and its *n*-butanol fraction exhibited a promising radical scavenging effect with EC_50_ values of 9.73 and 5.35 μg/mL for DPPH assay, and 0.338 and 0.271 μg/mL for ABTS test, respectively. The same study evidenced FRAP value is in the same range of potency of our sample [27].

Antioxidant activity may arise from a number of properties exhibited by major compounds such as catechin, sinapoyl-*O*-glucoside, quercetin-glycosides, bayogenin, and procyanidin isomer. Flavonoids such as catechin and quercetin derivatives have shown important protective activities, demonstrating the enhanced antioxidant effect in ethanol-treated rats and in H_2_O_2_-treated liver cells. Quercetin and catechin cooperatively inhibited IKKα/p53 pathway and activated the Nrf2 signaling pathway. IKKα was a critical negative regulator in their joint action [28]; sinapoyl-*O*-glucoside, a derivative of the sinapic acid, identified in various *Brassica* species, acted as an antioxidant by scavenging free radicals and reducing oxidative stress, including protection against oxidative stress-related diseases [29]; bayogenin isomers have instead demonstrated very important activities: in addition to the antioxidant activity [30], they have shown antimicrobial activity against bacterial and fungal strains *Bacillus subtilis*, *Escherichia coli*, *Mucor miehei*, and *Candida albicans* [30], and *in vitro* hepatoprotective effects against CCl_4_ induced toxicity [31].

### 2.3. Hypoglycaemic and Hypolipidemic Effect of Argania spinosa Husk Extracts

The extracts obtained from argan skin by-products were also assessed against enzymes involved in carbohydrate and fat digestion. All extracts exhibited enzyme inhibitory effects in a concentration-dependent manner. Acetone extract resulted in being the most active against both α-glucosidase and α-amylase enzymes with IC_50_ values of 12.37 and 18.93 µg/mL, respectively. Both values are lower than the positive control acarbose (Table 3, Figure S2a,b).

Methanol extract exhibited the highest lipase inhibitory activity with an IC_50_ value of 19.21 µg/mL. Previously, Daoudi et al. [32] investigated the effect of unroasted (UNR)/roasted(R) *A. spinose* seeds oil against carbohydrate-hydrolyzing enzymes (α-glucosidase and α-amylase). The results evidenced that both samples inhibited α-glucosidase with IC_50_ values of 0.081 and 0.117 mg/mL, for UNR and R samples, respectively. A similar effect was observed, also, α-amylase with IC_50_ values of 5.87 and 23.98 mg/mL for UNR and R samples, respectively. *In vivo* studies evidenced that the oral intake of both oils (2 mL/Kg) significantly attenuated the hyperglycemia induced by the sucrose and the starch in the normal and STZ-diabetic rats.

In agreement with these results, Kamal et al. [33] demonstrated that saponin-rich extract obtained from argan cake by-products was able to inhibit carbohydrate-hydrolysis enzymes with IC_50_ values of 209.10 and 0.89 mg/mL against α-amylase and α-glucosidase, respectively. Moreover, the extract significantly reduced blood glucose concentration in diabetic mice with activity comparable to metformin. The effect of *Argan* fruit methanol extract against α-glucosidase (EC_50_ values of 0.15 vs. 0.23 mg/mL) was also demonstrated [26].

## 3. Materials and Methods

### 3.1. Plants Material

**Figure 2:**
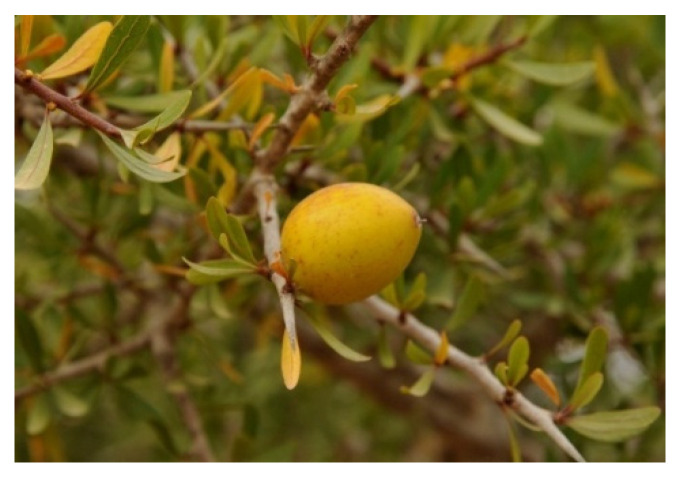
*Argania spinosa* (L.) Skeel fruit. Photo by Prof. Vincenzo Ilardi.

*Argania spinosa* (L.) Skeels fruits were collected near Ounagha, Morocco, 31°31′39″ N, 9°33′41″ O 242 m s/l. in May 2023 (Figure 2). Authentication was performed by Prof. Vincenzo Ilardi, and a voucher specimen has been deposited in the University of Palermo (reference number PAL109781).

### 3.2. Extraction of Plant Material

**Figure 3:**
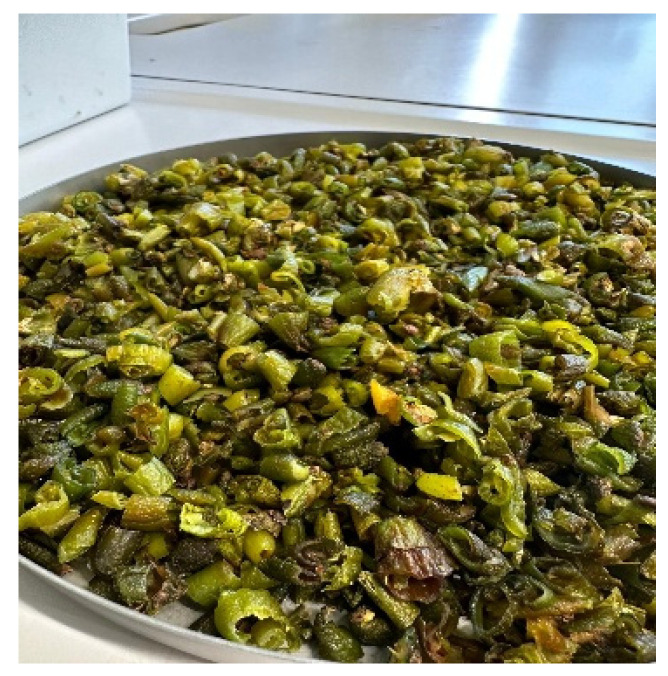
*Argania spinosa* (L.) husks. Photo by Dr. Natale Badalamenti.

Fresh fruits of *Argania spinosa* (1.2 kg) were manually peeled, and the husk (420 g) was manually removed (Figure 3), freeze-dried to give 225 g of dry material, and extracted successively with *n*-hexane (Merck, Milan, Italy), for one week (800 mL × 3-times), acetone (Merck, Milan, Italy) (800 mL × 3-times), and methanol (Merck, Milan, Italy) (800 mL × 3-times). The resulting solutions were evaporated to give 3.8 and 2.2 g for the acetone and methanol extracts, respectively.

### 3.3. LC-ESI/HRMS/MS Analysis

Qualitative UHPLC-(−)ESI/Q Exactive MS/MS analysis was conducted using an ultra-high-performance liquid chromatography system (UltiMate 3000 UHPLC, Dionex, Sunnyvale, CA, USA) coupled to an electrospray ionization source and a high-resolution mass spectrometer (Q Exactive Hybrid Quadrupole-Orbitrap, Thermo Fisher Scientific, Waltham, MA, USA). Chromatographic separations were performed on a Luna 5 µm C18(2) 100 Å column (150 mm × 2.1 mm; Phenomenex, Aschaffenburg, Germany) at a flow rate of 0.2 mL/min. A binary solvent system was employed, consisting of eluent A (water with 0.1% formic acid, Merck, Milan, Italy) and eluent B (acetonitrile with 0.1% formic acid, Merck, Milan, Italy). The column temperature was maintained at 30 °C throughout the separation (45 min) and re-equilibration (10 min) phases. The gradient elution program included a linear increase from 5% to 95% B over 30 min, held at 95% B for 5 min, followed by a return to 5% B over 5 min. The autosampler was set to inject 5 µL of the extracts (1.0 mg/mL), with each sample analyzed in triplicate. In negative ion mode, the electrospray ionization (ESI) source parameters were set as follows: sheath gas at 50 arbitrary units, auxiliary gas at 10 arbitrary units, and capillary temperature at 300 °C. The mass spectrometer operated over a mass range of *m*/*z* 120–1600, with a resolution of 70,000 and an automatic gain control (AGC) target of 3 × 10^6^ [21]. Data-dependent acquisition (DDA), with a resolution of 17,500, was employed. MS/MS spectra were acquired for the five most intense ions from the high-resolution MS scan, using a normalized collision energy of 30%, a minimum signal threshold of 3 × 10^4^, and an isolation window of 2.0 *m*/*z* [21].

### 3.4. Total Phenolic Content (TPC) and Total Flavonoid Content (TFC)

The Total Phenolic Content (TPC) was assessed using the Folin–Ciocalteu method as previously described [12]. Briefly, extract was mixed with Folin–Ciocalteu reagent, sodium carbonate, and distilled water. After 2 h of incubation at room temperature, the absorbance was read at 765 nm by using the UV-Vis Jenway 6003 spectrophotometer (Nottingham, UK). Results were reported as mg of chlorogenic acid equivalent (CAE) equivalents/g dry extract.

For Total Flavonoid Content (TFC), the extract was mixed with sodium nitrite and distilled water. After incubation for 5 min at room temperature, aluminum chloride was added. After 5 min, sodium hydroxide 1 M and water were added. The absorbance was read at 510 nm by using the UV-Vis Jenway 6003 spectrophotometer (Nottingham, UK). Results were reported as mg quercetin equivalents (QE)/g dry extract [12].

### 3.5. Antioxidant Activity

#### 3.5.1. Radical Scavenging Potential

The radical scavenging activity was assessed by 2,2′-azino-bis (3-ethylbenzothiazoline-6-sulfonic acid) diammonium salt) (ABTS) and 2,2-diphenyl-1-picrylhydrazyl (DPPH) tests.

For the ABTS test, the procedure previously reported was performed [12]. In this test the ABTS^·+^ radical cation solution with an absorbance value of 0.70 ± 0.03 nm at 734 nm was added to the extract (25 µL) at different concentrations (5–400 µg/mL) and left to react for 6 min at 25 °C. After that, the absorbance was read at 734 nm by using the UV-Vis Jenway 6003 spectrophotometer (Nottingham, UK).

In the DPPH test, extracts at different concentrations (62.5–1000 µg/mL) were mixed with DPPH solution (1.0 × 10^−4^ M), and after 30 min, the absorbance was read at 517 nm by using the UV-Vis Jenway 6003 spectrophotometer (Nottingham, UK) [12].

Ascorbic acid was used as positive control in both tests. Results are expressed as the concentration of extract at which the neutralization of free radicals is 50% (IC_50_) values (µg/mL).

#### 3.5.2. *β*-Carotene Bleaching Test

For the *β*-carotene bleaching test, a mixture of *β*-carotene, linoleic acid, and Tween 20 was prepared. Extracts at different concentrations (2.5–100 µg/mL) were mixed with emulsion and left to react for 30 and 60 min. The absorbance was read at 470 nm by using the UV-Vis Jenway 6003 spectrophotometer (Nottingham, UK) against a blank [12]. Propyl gallate was the positive control. Results are reported as IC_50_ (µg/mL).

#### 3.5.3. FRAP Test

In the Ferric Reducing Ability Power (FRAP) test, tripyridyltriazine (TPTZ) reagent, FeCl_3_, HCl, and acetate buffer were mixed to obtain the FRAP solution. Then the extract (2.5 mg/mL) was added to the diluted FRAP solution. After 30 min of incubation at 25 °C, the absorbance was measured at 595 nm by using the UV-Vis Jenway 6003 spectrophotometer (Nottingham, UK) [12]. Butylated hydroxytoluene (BHT) was used as a positive control. The FRAP value was expressed as µM Fe (II)/g.

### 3.6. Carbohydrate Hydrolyzing Enzymes Inhibitory Activities

The *α*-amylase and *α*-glucosidase inhibitory activity was assessed as previously reported [12]. In the *α*-amylase inhibitory test, the enzyme (EC 3.2.1.1), starch, and colorimetric reagent (CRS) were mixed. After that, samples at different concentrations were added and left to react with the enzyme at room temperature for 5 min. The absorbance was read at 540 nm by using the UV-Vis Jenway 6003 spectrophotometer (Nottingham, UK).

In the α-glucosidase inhibitory test, maltose solution, α-glucosidase (EC 3.2.1.20), O-dianisidine (DIAN), and peroxidase/glucose oxidase (PGO) system-color reagent solution were prepared. Samples, at different concentrations, were mixed with maltose solution and enzyme solution. The resulting solution was left to react at 37 °C for half an hour min. To arrest the reaction, perchloric acid solution was added, and after centrifugation for 10 min at 3000× *g*, the supernatant was collected and mixed with DIAN and PGO and left to incubate at 37 °C for half an hour. The absorbance was read at *λ* = 492 nm by using the UV-Vis Jenway 6003 spectrophotometer (Nottingham, UK). Acarbose was used as a positive control in both tests.

### 3.7. Lipase Inhibitory Activity Test

The porcine pancreatic lipase inhibitory activity was determined following the procedure previously described [12]. Briefly, a solution of 4-nitrophenyl octanoate (NPC) in dimethyl sulfoxide (DMSO), lipase enzyme (EC 3.1.1.3), and Tris-HCl buffer (pH 8.5) was prepared. Samples, at different concentrations, were mixed with enzyme, NPC solution, and Tris-HCl buffer and left to react at 37 °C for half hour. After that the absorbance was read at *λ* = 405 nm by using the UV-Vis Jenway 6003 spectrophotometer (Nottingham, UK). Orlistat was used as a positive control.

### 3.8. Statistical Analysis

Samples were analyzed in triplicate. Results were expressed as the mean ± standard deviation (S.D.) (n = 3). Prism GraphPad Prism version 4.0 for Windows (GraphPad Software, San Diego, CA, USA) was used to calculate the concentration giving 50% neutralization/inhibition (IC_50_). Tukey’s test was used to determine any significant difference on chemical parameters among investigated samples.

## 4. Conclusions

This work offers an in-depth look at the phytochemistry, antioxidant, hypoglycemic, and lipase inhibitory properties of *Argania spinosa* (L.) husks. Argan oil is known for its medicinal properties, and in recent years, the potential offered by both the leaves and the fruit shells of this tree has been valorized. Little or no scientific research has been conducted on the husk. Forty-three compounds divided into different chemical classes, such as hydroxycinnamic acid derivatives, flavanol derivatives, saponins, and triterpenic acids, were identified.

Among them, several flavonoids and saponins (**1**, **4**, **5**, **8**–**14**, **19**, **21**, **30**), previously identified only in the Argan fruits, have been identified here in the husk for the first time. The occurrence of triterpenes like mediagenic acid and zahnic acid has been highlighted here for the first time in *A. spinosa.*

The husk acetone extract exhibited the highest inhibitory activity against carbohydrate-hydrolyzing enzymes, whereas methanol extract exhibited the most interesting activity against pancreatic lipase.

These results pointed out that this Argan by-product is still a rich source of bioactive phytochemicals with potential use in pharmaceutical, cosmetic, and food industries for the development of agents for the prevention of type 2 diabetes and obesity.

However, further *in vivo* studies are still necessary to confirm the by-product *in vitro* activity.

## Figures and Tables

**Table 1:** Metabolites identified in *A. spinosa* husk extracts by UHPLC-(−)ESI/Q Exactive MS/MS analysis.

No.	Name	R*_t_*	Delta ppm	Molecular Formula	[M-H]^−^	[(M + FA)-H]^−^	MS/MS	Methanol Extracts	Intensity in methanol Extract	Acetone Extract	Intensity in Acetone Extract
**1**	procyanidin B1	9.05	2.61	577.1356	C_30_H_26_O_12_		289.0714, 407.0765	X	3.16 × 10^6^	-	-
**2**	coumaroyl-*O*-glucoside	9.62	1.07	325.0929	C_15_H_18_O_8_		145.0283	X	1.81 × 10^6^	X	1.51 × 10^6^
**3**	caffeoyl-*O*-glucoside	9.70	3.64	341.0879	C_15_H_18_O_9_		161.0340	X	5.0 × 10^4^	-	-
**4**	catechin	9.76	3.75	289.0717	C_15_H_14_O_6_		179.0334, 203.0704, 245.0814	X	2.15 × 10^6^	X	9.32 × 10^4^
**5**	procyanidin B2	9.88	1.87	577.1351	C_30_H_26_O_12_		289.0714, 407.0765	X	9.25 × 10^6^	-	-
**6**	feruloyl-*O*-glucoside	10.03	2.82	355.1034	C_16_H_20_O_9_		175.0390	X	2.71 × 10^4^	X	4.61 × 10^5^
**7**	sinapoyl-*O*-glucoside	10.18	3.63	385.1143	C_17_H_22_O_10_		205.0497	X	9.27 × 10^5^	X	8.96 × 10^5^
**8**	epicatechin	10.41	2.47	289.0714	C_15_H_14_O_6_		179.0336, 203.0709, 245.082	X	2.66 × 10^7^	X	1.88 × 10^6^
**9**	procyanidin C1	10.55	−1.79	865.1994	C_45_H_38_O_18_		289.0714, 407.0765	X	5.46 × 10^5^	-	-
**10**	rutin arabinoside	10.68	2.28	741.1889	C_32_H_38_O_20_		301.0329	X	1.34 × 10^6^	X	2.40 × 10^6^
**11**	quercetin glucoarabinoside	11.16	2.11	595.1306	C_26_H_28_O_16_		301.0472, 445.0772	X	1.03 × 10^6^	X	1.26 × 10^6^
**12**	quercetin-3-*O*-rhamnoglucoside	11.54	2.53	609.1465	C_27_H_30_O_16_		301.0376, 463.0874	X	7.23 × 10^6^	X	7.96 × 10^6^
**13**	quercetin-3-*O*-glucoside	12.01	2.76	463.0884	C_21_H_20_O_12_		301.0344	X	1.64 × 10^7^	X	4.40 × 10^7^
**14**	quercetin-3-*O*-arabinoside	12.71	1.97	433.07724	C_20_H_18_O_11_		301.0347	X	9.25 × 10^6^	X	6.60 × 10^7^
**15**	3-*O*-*β*-[*β*-D-glucopyranosyl-(1->3)-*β*-D-glucopyranosyl]-28-*O*-{α-L-rhamnopyranosyl-(1->3)-*β*-D-xylopyranosyl-[(1->f4)-α-L-rhamnopyranosyl-(1->2)-α-L-arabinopyranosyl}-16α-hydroxyprotobassic acid.	13.87	−0.69	1545.6945	C_70_H_114_O_37_		483.1714, 519.3321, 843.4385	X	4.56 × 10^6^	X	2.98 × 10^4^
**16**	arganine A	14.15	−1.70	1399.6361	C_64_H_104_O_33_		519.3326, 843.4382	X	3.09 × 10^7^	X	7.60 × 10^5^
**17**	arganine B	14.31	−0.19	1385.6217	C_63_H_102_O_33_		469.1568, 519.3329, 843.4392	X	2.30 × 10^5^	X	5.21 × 10^4^
**18**	arganine C	14.32	0.04	1237.5848	C_58_H_94_O_28_		519.3343, 681.3847	X	9.26 × 10^4^	X	4.91 × 10^4^
**19**	quercetin glycosinapate	14.45	2.39	669.1466	C_32_H_30_O_16_		301.0342, 463.0880	X	3.60 × 10^5^	X	4.98 × 10^6^
**20**	butyroside B	14.50	0.56	1223.5698	C_57_H_92_O_28_		519.3328, 681.3845	X	1.33 × 10^5^	-	-
**21**	quercetin glycocoumarate	14.67	1.84	609.1250	C_30_H_26_O_14_		301.0350, 463,0879	X	2.83 × 10^6^	X	1.22 × 10^7^
**22**	3-*O*-*β*-[*β*-D-glucopyranosyl-(1->3)-*β*-D-glucopyranosyl]-28-O-{α-L-rhamnopyranosyl-(1->3)-*β*-D-xylopyranosyl-[(1->3)-α-L-rhamnopyranosyl]-(1->4)-R-L-rhamnopyranosyl-(1->2)-α-Larabinopyranosyl] protobassic acid.	15.00	−0.03	1529.7036	C_70_H_114_O_36_		483.1722, 503.3382, 827.4445	X	1.63 × 10^6^	-	-
**23**	arganine D	15.22	−0.88	1383.6415	C_64_H_104_O_32_		503.3375, 665.3900, 827.4495	X	2.34 × 10^7^	X	2.12 × 10^5^
**24**	arganine E	15.33	0.64	1369.6279	C_63_H_102_O_32_		503.3375, 665.3912	X	2.05 × 10^6^	-	-
**25**	arganine J	15.42	0.37		C_62_H_100_O_30_	1369.6235	487.2912	X	1.17 × 10^6^	-	-
**26**	mi-saponin A	15.47	0.01	1221.5898	C_58_H_94_O_27_		503.3382, 665.3906	X	1.99 × 10^6^	-	-
**27**	arganine G	15.55	−1.58		C_47_H_76_O_19_	989.4971	487.3105	X	1.12 × 10^5^	X	5.77 × 10^4^
**28**	arginine F	15.66	2.02	1207.5767	C_57_H_92_O_27_	1253.5797	503.3325, 665.3908	X	1.06 × 10^6^	-	-
**29**	butyroside C	16.13	0.95	1235.5703	C_58_H_92_O_28_		503.3365, 679.3692,	X	5.64 × 10^6^	X	1.42 × 10^5^
**30**	quercetin	16.16	3.82	301.0354	C_15_H_10_O_7_		151.003, 178.9977	X	1.74 × 10^5^	X	5.69 × 10^6^
**31**	zanhic acid	17.69	0.71	517.3163	C_30_H_46_O_7_		499.3049	-	-	X	5.52 × 10^6^
**32**	16-α-Hydroxyprotobassic acid	17.89	1.71	519.3325	C_30_H_48_O_7_		457.3333, 501.3228	-	-	X	7.56 × 10^6^
**33**	medicagenic acid	18.24	2.14	501.3221	C_30_H_46_O_6_		425.3045, 483.3114	-	-	X	1.22 × 10^7^
**34**	protobassic acid	18.69	1.60	503.3375	C_30_H_48_O_6_		453.3019, 485.3273	-	-	X	5.42 × 10^6^
**35**	2,3-dihydroxy-30-norolean-12,20(29)-dien--23,28-dioic acid	19.09	2.32	485.2909	C_29_H_42_O_6_		425.2694, 467.2805	-	-	X	6.52 × 10^6^
**36**	medicagenic acid isomer	19.30	1.30	501.3217	C_30_H_46_O_6_		441.3011, 483.3122	-	-	X	3.80 × 10^7^
**37**	protobassic acid isomer	19.72	1.66	503.3375	C_30_H_48_O_6_		441.3369, 485.3273	-	-	X	6.80 × 10^6^
**38**	2,3-dihydroxy-23-oxo-30-norolean-12,20(29)-dien-28-oic acid	21.11	2.62	469.2961	C_29_H_42_O_5_		451.2854	-	-	X	2.14 × 10^6^
**39**	bassic acid	21.60	2.61	485.3274	C_30_H_46_O_5_		467.3168	-	-	X	6.20 × 10^6^
**40**	bayogenin	22.02	1.37	487.3425	C_30_H_48_O_5_		409.3071, 457.3319	-		X	1.97 × 10^7^
**41**	bayogenin isomer	22.46	2.17	487.3429	C_30_H_48_O_5_		469.3329	-	-	X	8.52 × 10^6^
**42**	maslinic acid	28.06	1.70	471.3477	C_30_H_48_O_4_		359.8896	-	-	X	4.48 × 10^6^
**43**	oleanolic acid	32.95	2.13	455.3529	C_30_H_48_O_3_			-	-	X	5.25 × 10^5^

**Table 2:** Antioxidant potential of *Argania spinosa* (L.) husk CH_3_OH and (CH_3_)_2_CO extracts.

Extract	FRAP Test	*β*-Carotene Bleaching Test		DPPH Test	ABTS Test
		t = 30 min	t = 60 min		
	μM Fe (II)/g	IC_50_ (μg/mL)	IC_50_ (μg/mL)	IC_50_ (μg/mL)	IC_50_ (μg/mL)
CH_3_OH	54.88 ± 8.32 ^a^	41.36 ± 5.23 ^b^	29.14 ± 3.70 ^b^	198.34 ± 12.67 ^b^	302.23 ± 14.71 ^a^
(CH_3_)_2_CO	36.37 ± 4.06 ^b^	26.68 ± 3.64 ^a^	13.82 ± 2.40 ^a^	226.77 ± 13.01 ^a^	436.33 ± 15.90 ^b^
Sign.	**	**	**	**	**
Ascorbic acid,	-	-	-	5.03 ± 0.82	1.78 ± 0.13
BHT	63.44 ± 2.86				
Propyl gallate		1.04 ± 0.05	0.09 ± 0.07		

Data are reported as mean standard deviation (SD) (*n* = 3). Differences between samples were evaluated by one-way ANOVA followed by Tukey’s test. Results followed by different letters are significantly different at ** *p* < 0.01.

**Table 3:** Hypoglycaemic and hypolipidemic effect of *Argania spinosa* (L.) husk CH_3_OH and (CH_3_)_2_CO extracts.

Extract	*α*-Amylase	*α*-Glucosidase	Lipase
	IC_50_ (μg/mL)	IC_50_ (μg/mL)	IC_50_ (μg/mL)
CH_3_OH	25.24 ± 3.90 ^b^	27.42 ± 4.11 ^b^	19.21 ± 5.05 ^a^
(CH_3_)_2_CO	18.93 ± 2.68 ^a^	12.37 ± 3.66 ^a^	23.67 ± 4.41 ^b^
Sign.	*	**	*
Acarbose	52.75 ± 1.25	35.43 ± 1.12	-
Orlistat	-	-	37.87 ± 1.21

Data are reported as mean standard deviation (SD) (*n* = 3). Differences between samples were evaluated by one-way ANOVA followed by Tukey’s test. Results followed by different letters are significantly different at ** *p* < 0.01; * *p* < 0.05.

## Data Availability

All data and materials are available upon request from the corresponding author.

## Supplementary Materials

The following supporting information can be downloaded at: https://www.mdpi.com/article/10.3390/plants14152288/s1, Figure S1. Radical scavenging potential assessed by ABTS (a) and DPPH (b) test by *Argania spinosa* husk MeOH and acetone extracts; Figure S2. Percentage of inhibition of *A. spinosa* husk’s extracts in *β*-carotene bleaching test at (a) 30 min incubation (b) 60 min incubation.
