# Supplementary material for: Phytochemical Profile and Functional Properties of the Husk of Argania spinosa (L.) Skeel
Source: Plants (Basel). 2025 Jul 24;14(15):2288. doi: 10.3390/plants14152288 (PMC12348269; doi:10.3390/plants14152288)
Supplement: Supplementary file 1 [file plants-14-02288-s001.zip › plants-3768962-supplementary.pdf]

Supplementary Materials

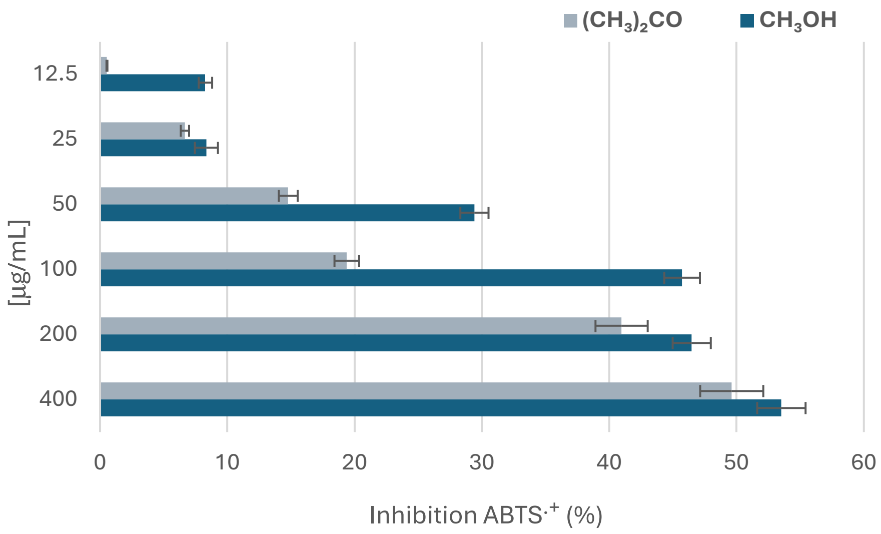

(a)

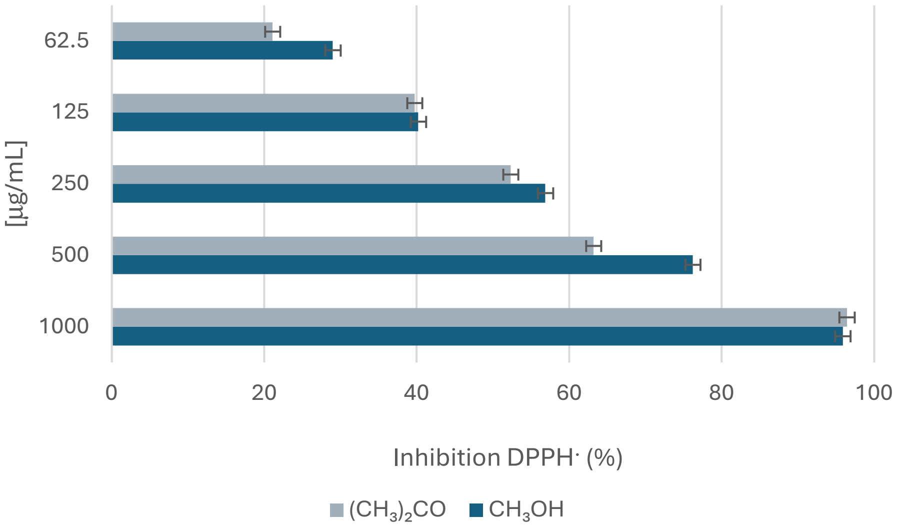

(b)

Figure S1. a and **b.** Radical scavenging potential assessed by ABTS (a) and DPPH (b) test by *Argania spinosa* husk MeOH and acetone extracts.

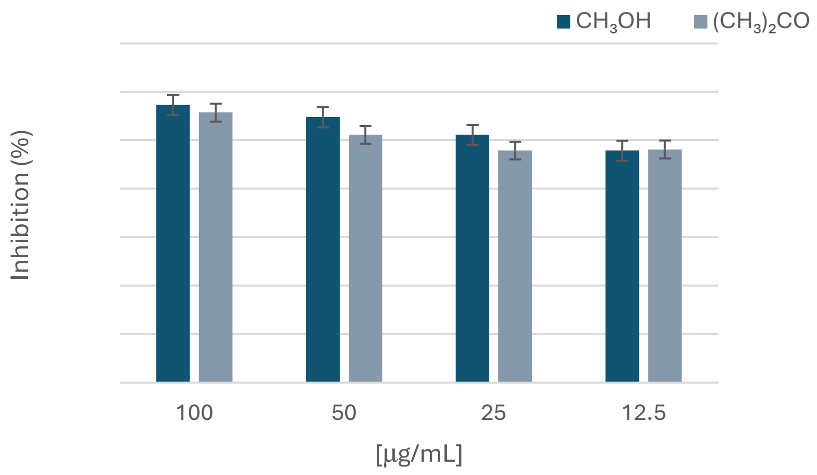

(a)

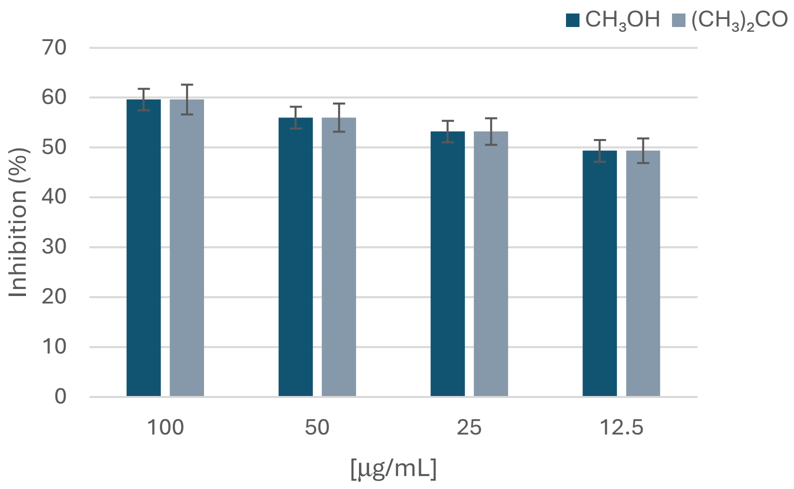

(b)

Figure S2. a and **b.** Percentage of inhibition of *A. spinosa* husk's extracts in β-carotene bleaching test at (a) 30 min incubation (b) 60 min incubation.
